# Orthodontic Treatment with Fixed Appliances Versus Aligners: An Experimental Study of Periodontal Aspects

**DOI:** 10.3390/dj13020070

**Published:** 2025-02-04

**Authors:** Lucia Giannini, Guido Galbiati, Francesco Carlo Tartaglia, Maria Elena Grecolini, Cinzia Maspero, Roberto Biagi

**Affiliations:** 1 Dipartimento di Scienze Biomediche, Chirurgiche e Odontoiatriche, Università degli Studi di Milano, 20122 Milan, Italy; lucia.giannini@unimi.it (L.G.); cinzia.maspero@unimi.it (C.M.); roberto.biagi@unimi.it (R.B.); 2 Fondazione IRCCS Cà Granda Ospedale Maggiore Policlinico, 20122 Milan, Italy; 3 Department of Biomedical Sciences, Humanitas University, Via Rita Levi, Montalcini 4, 20072 Pieve Emanuele, Italy; 4 Dipartimento Chirurgico Medico Odontoiatrico e di Scienze Morfologiche, Unità Complessa di Odontoiatria e Chirurgia Maxillo Facciale, Università degli Studi di Modena e Reggio Emilia, 41121 Modena, Italy; dott.elena@grecoliniortodonzia.it

**Keywords:** orthodontics appliances, oral hygiene, periodontal status

## Abstract

**Objective:** This study aimed to compare the effects of orthodontic treatment on the same patients using aligners (upper arch) versus traditional fixed appliances (lower arch) on oral hygiene and periodontal health. **Materials and Methods:** A total of 90 patients, all treated by the same orthodontist and with an average age of 26 years, were included in the study. The research focused on factors predisposing patients to periodontitis, as well as plaque and tartar accumulation. Statistical analysis was performed using the paired-samples Student’s t-test, with values of *p* < 0.05 deemed statistically significant. **Results:** Patients treated with fixed orthodontic appliances generally showed increased plaque accumulation (+10% PI) and a decline in periodontal health compared to those treated with aligners (40% presented a high level of S.Mutans and Lactobacilli). In patients who did not maintain proper oral hygiene, the initial conditions of the upper arch remained stable, while the lower arch exhibited significant deterioration, with increased plaque buildup and greater tissue inflammation. **Conclusions:** The findings emphasize the critical role of individual oral hygiene practices during orthodontic treatment in preserving periodontal health. Aligners appear to be more favorable in protecting periodontal tissues, improving plaque control, and reducing gingival inflammation. Various supplementary approaches for plaque management and microbial colonization in saliva should be implemented based on the type of orthodontic appliance used.

## 1. Introduction

Orthodontics plays a crucial role in improving bite abnormalities and aligning teeth, thereby enhancing the overall esthetic balance of the face and lips, as well as optimizing chewing functions and preserving both teeth and supporting tissues. The ultimate goal of orthodontic treatment is to achieve a harmonious smile while ensuring long-term oral health. Orthodontic appliances, whether fixed, removable, or hybrid, are commonly used to achieve these objectives [1]. However, despite their benefits, orthodontic appliances complicate oral hygiene practices, as they retain plaque and food debris, which increases the risk of tooth enamel damage and periodontal issues. Studies have shown that periodontal problems, such as gingival inflammation, plaque accumulation, and bleeding on probing, are particularly evident in patients treated with traditional fixed appliances [2].

In 1999, a new generation of orthodontic treatment emerged in the form of removable aligners. These aligners cover all teeth and part of the gum line, and they are gradually adjusted to move teeth into their desired positions. In addition to their esthetic appeal, aligners offer significant advantages over traditional braces. They are removable, allowing patients to maintain their usual oral hygiene habits and reducing the risk of inflammation in periodontal tissues. However, despite their advantages, no studies have yet compared fixed appliances and aligners in the same patient cohort [3,4].

Fixed orthodontic appliances, such as braces, require a more detailed approach to oral hygiene to prevent oral health complications. Orthodontic patients typically undergo monthly checkups, and specific oral hygiene guidelines are provided. It is essential for patients to follow proper dietary instructions, as a nutritious diet contributes to better oral health. Foods that are sticky or difficult to chew, like stringy meats, should be avoided as they can leave residue that is hard to remove. Without proper plaque removal, two major issues may arise: periodontal damage and enamel demineralization. Marginal gingivitis, which can progress to deep periodontal lesions, and white spot lesions are common consequences of neglecting oral hygiene during orthodontic treatment.

Dental plaque is composed of microbial communities that form biofilms on the tooth surface. These biofilms are more complex than the sum of their microbial species, and bacteria such as *Streptococcus mutans*, *Streptococcus sanguis*, and *Streptococcus salivarius* play a significant role in tooth decay and periodontal disease. The presence of orthodontic appliances exacerbates the situation by making it easier for bacteria to adhere to teeth and creating an environment conducive to biofilm formation. This highlights the importance of controlling plaque and inflammation before beginning orthodontic treatment [5].

To ensure effective prevention, orthodontic patients follow a four-phase approach: pre-treatment, treatment, post-treatment, and maintenance. During the pre-treatment phase, a preventive program is established, including proper food hygiene, regular professional cleaning, fluoride treatment, and groove sealing. Studies have shown that fluoride-containing bonding systems can help prevent enamel demineralization caused by orthodontic brackets. Patients must also adhere to personalized care plans that take into ac-count their periodontal tissues and enamel sensitivity [6].

During the active treatment phase with fixed appliances, oral hygiene becomes more complicated. Various elements of the fixed appliance, such as the brackets and bands, create spaces where plaque can accumulate. Preventing plaque buildup requires careful attention to appliance fitting, especially when using orthodontic bands. The orthodontist must ensure that the bands fit precisely and excess cement is removed, as improper fitting increases the risk of plaque accumulation and periodontal damage.

Removable orthodontic appliances, such as transparent aligners, have gained popularity due to their esthetic benefits and their ability to facilitate better oral hygiene compared to fixed braces. Aligners are custom designed to fit the patient’s teeth, and they can be adjusted over time to achieve the desired tooth movement. Since aligners can be removed, patients can maintain their usual oral hygiene practices, including brushing and flossing, without the obstruction of fixed appliances. This reduces the risk of plaque buildup and periodontal inflammation [7].

However, maintaining good oral hygiene with aligners requires high levels of patient compliance. Aligners must be worn consistently, and patients need to clean them regularly. Failure to follow these guidelines can lead to bacterial growth on the aligners, increasing the risk of oral health issues. In an effort to establish best practices for aligner care, studies have been conducted to evaluate the effectiveness of different cleaning protocols. One study tested three cleaning methods on thermoplastic aligners worn by patients for several weeks. The aligners were subjected to regular cleaning with fluoride toothpaste, a chlorhexidine mouthwash soak, and a sonic bath with a cleaning-crystal solution. The results showed that the sonic bath system was the most effective at reducing bacterial adhesion and plaque accumulation.

The increasing number of adult patients undergoing orthodontic treatment has raised concerns about the potential impact of orthodontic appliances on periodontal health. Multidisciplinary collaboration among orthodontists, periodontists, and dental hygienists is essential for optimizing treatment outcomes and preventing periodontal complications. Common orthodontic problems seen in periodontally compromised patients include anterior tooth malalignment, irregular interdental spaces, rotations, migrations, tooth loss, and traumatic bites. These conditions often arise from poor oral hygiene, leading to plaque accumulation and the onset of periodontal disease [8,9].

Orthodontic appliances can improve both the esthetic and functional aspects of a patient’s oral health. However, if not properly managed, these appliances can worsen periodontal conditions. Mechanical irritation caused by poorly fitting appliances and poor oral hygiene practices can contribute to gingival inflammation, plaque buildup, and the transformation of subgingival plaque into more aggressive bacterial flora. This progression from gingivitis to periodontitis underscores the importance of thorough oral hygiene before, during, and after orthodontic treatment.

Research has shown that with adequate plaque control, patients with compromised periodontal health can successfully undergo orthodontic treatment without further damaging their periodontal tissues. Conversely, inadequate oral hygiene during treatment can accelerate periodontal disease progression, particularly in patients with pre-existing periodontal issues. Studies have emphasized the importance of frequent plaque removal and proper care of orthodontic appliances to avoid exacerbating periodontal problems [10,11].

While fixed orthodontic appliances remain the gold standard for controlling tooth movement, in the case of dilacerated or impacted teeth, aligners have become increasingly popular for their esthetic benefits. Aligners are less invasive and generally have a positive impact on periodontal health, with no significant risk of enamel demineralization or root resorption compared to traditional fixed appliances. Studies have shown that aligner treatment can improve periodontal conditions, reduce soft tissue lesions, and alleviate temporomandibular joint (TMJ) disorders [12,13].

However, the success of aligner treatment depends on patient compliance. If patients do not follow the recommended wear schedule, treatment efficacy may be compromised. Additionally, aligners may not be suitable for all types of orthodontic movements, particularly in cases that require significant tooth movement or complex corrections. In such cases, orthodontists may integrate aligners with fixed appliances to achieve the desired results [13].

The aim of this study was to evaluate and compare orthodontic treatment outcomes using conventional fixed appliances and aligners with respect to oral hygiene and periodontal health. A key innovation of this research lied in the simultaneous application of the two treatment methods on the same patients: aligners were used in the maxillary arch and fixed appliances in the mandibular arch of 90 participants. This unique study design allowed for a direct comparison of the two systems both cross-sectionally across all patients and individually within each patient, eliminating inter-patient variability. 

This study was funded by Italian Ministry of Health—Current Research IRCCS.

## 2. Materials and Methods

A total of 90 patients were selected for this study, including 43 females and 47 males, with a mean age of 26 years. All participants were at least 18 years old. To avoid false positives or false negatives, exclusion criteria were applied based on predisposition to periodontal disease and plaque and tartar accumulation in order to standardize the study group. Specifically, the exclusion criteria, derived from risk factors most supported by scientific evidence, were as follows:

- Smokers: Smoking is a major risk factor for periodontitis. Smokers are significantly more likely to develop periodontal disease and respond less effectively to periodontal treatment than non-smokers.
- Diabetics (type 1 and type 2): Epidemiological data suggest that diabetes contributes to the development of periodontal lesions.
- Obese patients: Obesity promotes systemic inflammation, alters lipid metabolism, and induces insulin resistance.
- Patients with HIV: Progressive immune dysfunction is closely associated with periodontitis.
- Patients with hormonal changes: Hormonal fluctuations during pregnancy and menopause significantly affect the gingiva, increasing the risk of periodontal disease. The gingiva is a target tissue for steroid hormone action, particularly estrogen, which influences collagen metabolism, angiogenesis, and interactions with inflammatory mediators.
- Patients with a family history of periodontitis: This criterion was used to rule out genetic predisposition.
- Patients undergoing specific drug therapies: Medications such as birth control pills, antidepressants, and oral antihypertensives, which can increase gingival volume, were excluded.
- Initially, a larger pool of patients was considered for this study. However, after applying the exclusion criteria, only 90 patients met the necessary requirements for participation. We believe that this sample size is sufficient for this first exploratory study, which is innovative in comparing the two orthodontic treatment methods (fixed appliances and aligners) within the same patients.

All patients included in the study presented mild-to-moderate dental crowding and had been in orthodontic treatment for less than one year. We proposed a system of transparent aligners for all patients with recognized indications and specific esthetic needs for social or relational reasons. The originality of our study lied in the inclusion of patients whose maxillary arches were treated with aligners, while their mandibular arches were simultaneously treated with fixed appliances [14,15].

The combined use of a fixed orthodontic appliance on the lower arch and removable aligners on the upper arch can be explained by different clinical needs, which are linked to the specific situation of the considered sample size.

Clinical reasons:

1. Differences in orthodontic problems between the upper and lower arches:

- The lower arch requires better control of tooth movements, such as rotation or intrusion, which could be better managed with fixed appliances.
- The upper arch has simpler tooth movements that aligners can achieve effectively.

2. Esthetics:

- The choice of aligners for the upper arch was dictated by esthetic needs to minimize the visibility of the appliance.

Furthermore, in some patients, special conditions of the upper arch, like the presence of prosthetic restorations, enamel problems, or other situations, made treatment with aligners a less invasive and more conservative solution.

The combination of different orthodontic techniques can be customized to patient needs, balancing treatment effectiveness, esthetics, and practicality. As far as oral hygiene is concerned, aligners offer an advantage in terms of ease of cleaning compared to fixed braces but still require a careful routine for both the teeth and the aligners themselves.

Following the initial consultation, orthopantomography (OPT) and lateral teleradiography were performed and dental impressions were taken for case study preparation. At the time of the first aligner delivery, which was concurrent with the placement of the fixed appliance (T0), all patients underwent a comprehensive periodontal assessment, evaluation of tissue health, professional oral hygiene, and home hygiene training based on specific protocols [14,15].

For the mandibular arch, a multi-bracket system (metal self-ligating brackets) utilizing the straight-wire technique was employed.

In this study, the presence of cocci and bacilli (both Gram-positive and Gram-negative) was evaluated at different stages of orthodontic treatment, distinguishing between the arch treated with aligners and the arch treated with fixed orthodontic appliances.

In the oral cavity, both cocci and bacilli can cause issues, but bacilli (such as *Streptococcus mutans* and *Lactobacilli*) are more commonly associated with dental diseases like caries. These bacteria are capable of metabolizing sugars, producing acids that demineralize tooth enamel and lead to cavities.

Cocci (such as *Staphylococcus aureus* and *Streptococcus pyogenes*) may also be present in the mouth, but they are generally not the primary contributors to caries. However, some cocci can be involved in gingival infections or other oral conditions, such as abscesses [14].

In summary, bacilli, particularly *S. mutans*, are generally considered more harmful to oral health due to their role in causing caries and periodontal disease. Nevertheless, maintaining a balanced oral microbiome is crucial, and following proper oral hygiene protocols helps prevent the accumulation of harmful bacteria of any kind.

Periodontal monitoring was performed at three time points:

- Appliance positioning (T0);
- One month after the start of treatment (T1);
- Five months after the start of treatment (T2);
- At the end of treatment (9–12 months) (T3).

Sampling was performed using subgingival plaque scraping from the mesiobuccal surface of every tooth. Bacterial quantification was carried out using the culture methods to measure specific bacterial markers and periodontal pathogens.

The protocol adhered to the periodontal guidelines of the Italian Society of Periodontology (SIDP) [15]. Patients with mobile teeth or severe periodontal disease were excluded from the study.

Statistical analysis was conducted using Student’s *t*-test for paired samples to compare the two treatment modalities (aligners in the maxillary arch and fixed appliances in the mandibular arch) within the same patients. This approach was chosen to account for the paired nature of the data and to evaluate intra-patient differences in oral hygiene and periodontal health outcomes. A *p*-value of <0.05 was considered statistically significant.

## 3. Results

**Figure 1:**
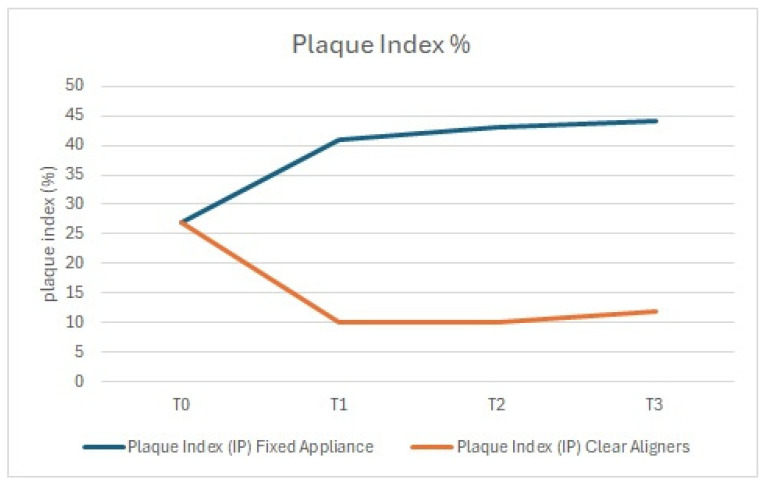
Plaque index: comparison between the arches treated with clear aligners and those treated with fixed appliances.

On the dental arches treated with aligners, after the initial motivation and instructions, the plaque index significantly decreased and was maintained over time (Figure 1), whereas in the arches treated with fixed orthodontic appliances, a statistically significant increase in PI was found (+10% at T1, T2, and T3). Furthermore, at T2, 40% of the arches treated with fixed appliances exhibited elevated levels of *S. mutans* and *Lactobacilli*, both of which are considered risk factors for oral health.

**Figure 2:**
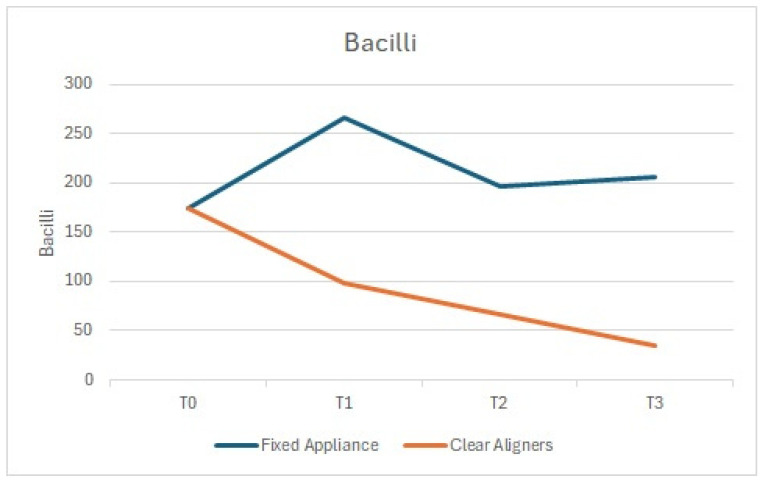
Bacilli: comparison between the arches treated with clear aligners and those treated with fixed appliances.

**Figure 3:**
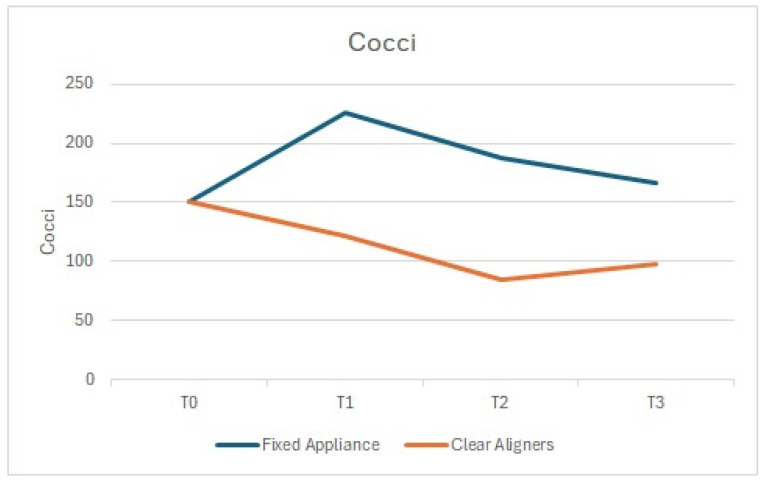
Cocci: comparison between the arches treated with clear aligners and those treated with fixed appliances.

Similarly, the number of cocci and bacilli showed a comparable trend, with a statistically significant reduction at T1 compared to T0 in the arches treated with clear aligners, and an increase in those treated with fixed appliances (Figure 2 and Figure 3).

**Figure 4:**
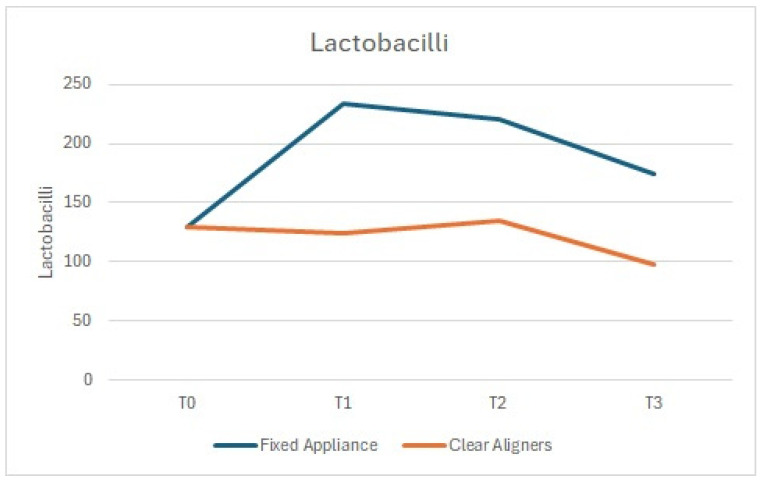
*Lactobacilli*: comparison between the arches treated with clear aligners and those treated with fixed appliances.

**Figure 5:**
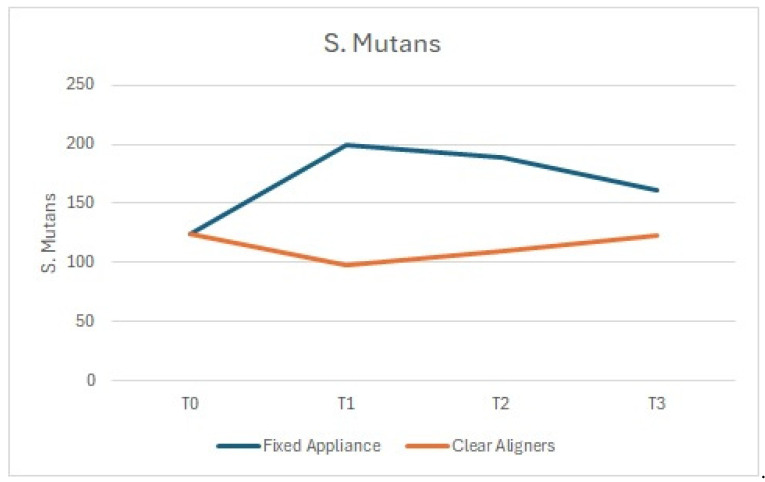
*S. mutans*: comparison between the arches treated with clear aligners and those treated with fixed appliances.

Gram-positive bacteria (including *S. mutans* and *Lactobacilli*), often associated with dental caries, showed a significant increase in the arches treated with fixed appliances compared to those treated with aligners (Figure 4 and Figure 5).

A similar trend was observed for Gram-negative bacteria (*Porphyromonas gingivalis*, *Treponema denticola*, and *Fusobacterium nucleatum*), which are more strongly associated with periodontal disease and capable of contributing to systemic pathologies.

The confidence interval for the results was calculated at 95%.

## 4. Discussion

For many years, the use of fixed orthodontic appliances has been linked to an increase in plaque accumulation and a deterioration of periodontal health, as evidenced by various periodontal disease indicators. Several studies have suggested that periodontal health tends to improve once these appliances are removed; however, it has also been noted that the subgingival flora shifts from aerobic Gram-positive species to more anaerobic Gram-negative species, such as *Prevotella*, which are commonly associated with periodontitis during orthodontic appliance use [16,17,18,19,20,21].

The periodontal status of patients treated with both fixed and removable orthodontic appliances has yielded conflicting results in different studies. While some authors report significant differences in periodontal outcomes between these two treatment methods, others have found no notable variations in the depth of probing or other clinical indicators.

A study conducted between February 2002 and August 2003 in Germany evaluated the periodontal health of patients treated with either fixed orthodontic appliances or the Invisalign system. The study, which took place at the Department of Orthodontics and Dentofacial Plastic Surgery of the Charité University of Berlin, followed 30 patients treated with fixed orthodontic appliances and 30 patients treated with aligners. Periodontal health was assessed through three evaluations during treatment, using indices such as the gingival index (GI), plaque index (PI), papillary bleeding index (PBI), and probing depth loss. These parameters were measured in all permanent teeth, from the central incisors to the first molars, and were recorded alternately at the vestibular and lingual/palatal sites in the maxillary and mandibular quadrants. Probing depth was assessed in four regions: mesial, distal, lingual, and vestibulo-lateral around the first molar and premolar of each quadrant. Detailed oral hygiene instructions were provided to each patient at every follow-up visit. The study included patients aged between 18 and 51 years who had been undergoing treatment for at least six months in both arches [22].

Gingival inflammation was assessed using Løe’s gingival index (GI) and Silness and Saxer’s papillary bleeding index (PBI), while plaque accumulation was evaluated through Løe and Silness’s plaque index (PI). The PBI was assessed on a five-point scale, with bleeding intensity observed upon probing. The PI was assessed on a four-point scale based on plaque accumulation at the gingival margin. To facilitate comparison, an average score was calculated to reflect the overall periodontal condition of each patient.

At the first evaluation, the results of the gingival index, papillary bleeding index, and probing depth loss were similar between the two treatment groups. The only significant difference at this time was the plaque index (PI), which favored the group treated with Invisalign. This finding suggests that fixed orthodontic appliances, which include brackets, bands, and arches, present greater challenges for maintaining oral hygiene compared to aligners, which can be removed for cleaning. However, all periodontal indicators showed some improvement from the first to the final visit, likely due to the individualized oral hygiene instruction provided at each appointment.

Interestingly, despite expectations, the Invisalign system did not demonstrate superior periodontal outcomes compared to traditional fixed appliances. This could be attributed to the fact that aligners cover the surface of the teeth for extended periods, potentially trapping food particles and other substances, which can lead to irritation, particularly in patients who consume sugary snacks and beverages. Additionally, the edges of aligners are not always perfectly smooth, which can lead to irritation of the gingival margins. Nevertheless, the use of aligners still showed some advantages, particularly in terms of reduced plaque buildup and the potential for more effective oral hygiene, as patients were able to remove the aligners to clean their teeth more thoroughly than with fixed appliances [22].

In a separate study involving 42 adult patients from New York University’s Department of Orthodontics, the periodontal status of individuals treated with either fixed orthodontic appliances or removable aligners was monitored over the course of one year. Of the 42 subjects, 22 received fixed appliances and 20 used removable aligners. The study evaluated periodontal health using indices such as the plaque index (PI), gingival index (GI), bleeding on probing (BOP), and probing depth (PPD). Additionally, plaque samples were analyzed using the BANA test, which detects specific bacteria in subgingival plaque that produce toxic sulfur metabolites and are associated with periodontitis.

The study’s findings revealed no significant differences in BANA test scores between the two groups at baseline or at six weeks, but after six months, patients with fixed orthodontic appliances exhibited a sevenfold higher probability of scoring positive for the presence of anaerobic bacteria such as *Treponema denticola*, *Porphyromonas gingivalis*, and *Tannerella forsythia*, all of which are implicated in periodontal disease. In contrast, patients treated with removable aligners showed better oral hygiene outcomes, as evidenced by lower plaque levels, less gingival bleeding, and improved periodontal health [23].

In conclusion, while fixed orthodontic appliances continue to pose challenges to maintaining optimal periodontal health, removable aligners such as the Invisalign system can offer some advantages, including easier oral hygiene management and improved periodontal outcomes. These findings suggest that aligners may be a more suitable option for adult patients at risk for periodontal disease, but continued emphasis on oral hygiene remains critical for all orthodontic patients [24,25,26,27,28].

The reduction in Gram-negative bacteria observed with aligners can be attributed to the superior ability of patients to maintain oral hygiene during treatment. Unlike fixed appliances, which include brackets and wires that create additional retention sites for plaque accumulation and make brushing and flossing more challenging, aligners are removable. This allows patients to perform regular and thorough oral hygiene practices, such as brushing and interdental cleaning, without obstruction. Consequently, the bacterial load, particularly that of Gram-negative periodontal pathogens, is more effectively controlled in patients treated with aligners.

## 5. Conclusions

Orthodontic treatments, whether with fixed appliances or aligners, can significantly improve a patient’s dental health and esthetic appearance. However, both treatment options pose challenges for maintaining proper oral hygiene, and patients must be vigilant in following hygiene protocols to prevent complications such as plaque buildup, gingival inflammation, and enamel demineralization. While aligners offer several advantages, including improved esthetics and easier maintenance of oral hygiene, patient compliance is critical for achieving successful outcomes. Ultimately, collaboration between orthodontists, periodontists, and dental hygienists is essential for ensuring the best possible results and minimizing the risks associated with orthodontic treatment.

The specificity of this study allowed for the first direct comparison between these two different orthodontic treatment methods, based on data collected from the same patients under the same basic biological conditions. This opens up interesting prospects for further research. According to the scientific literature, fixed orthodontic appliances are associated with greater plaque accumulation and poorer periodontal health compared to aligners. This was confirmed in all patients in our sample. The results also emphasize the importance of individual oral hygiene practices during orthodontic treatment. In patients who followed the recommended hygiene procedures, the maxillary arch treated with aligners showed improvement in all evaluated parameters, whereas only a slight deterioration was observed in the mandibular arch treated with fixed appliances. Conversely, in patients who did not maintain good hygiene, the initial condition remained unchanged in the maxillary arch, while a marked deterioration was observed in the mandibular arch, including plaque accumulation and increased tissue inflammation. Periodontal survey values were more stable throughout the study period, except in one case where probing depth increased due to gingival hyperplasia in the lower arch.

With effective bacterial plaque control, neither orthodontic treatment modality caused significant harm to the periodontal tissues. Aligners were a better choice for periodontal tissues, resulting in improved plaque accumulation levels and less gingival inflammation. Therefore, from a periodontal perspective, aligners represent the optimal treatment solution for cooperative adult patients and those with precise esthetic needs compared to fixed systems.

In this study, it was observed that clear aligners offer significant advantages in maintaining oral hygiene. However, it is important to also recognize the effectiveness of conventional fixed appliances, particularly the self-ligating metal bracket system, which remains a widely used and proven method in orthodontic treatment.

Various supplementary approaches for plaque management and microbial colonization in saliva should be implemented, depending on the type of orthodontic appliance used. For patients with fixed appliances, additional measures such as antimicrobial mouth rinses, special brushes for cleaning around brackets, and regular professional cleanings may be necessary to reduce plaque buildup. In contrast, for patients using removable aligners, it is crucial to emphasize the importance of regular cleaning of the aligners themselves, as well as consistent oral hygiene practices, to prevent bacterial accumulation.

This study has some limitations. First, the follow-up period was relatively short, ranging from 9 to 12 months, which may not be sufficient to fully assess the long-term effects of orthodontic treatment on oral hygiene and periodontal health. Additionally, the study’s reliance on a single operator for all procedures may introduce a bias, as the results could be influenced by the operator’s technique, experience, and consistency in treatment execution.

## Data Availability

The data presented in this study are available upon reasonable request, after the signature of a formal data-sharing agreement in an anonymous form, from the corresponding author because they are protected by privacy.

## Abbreviations

The following abbreviations are used in this manuscript:

MDPI	Multidisciplinary Digital Publishing Institute
DOAJ	Directory of open access journals
TLA	Three-letter acronym
LD	Linear dichroism
